# Pigment Epithelium-Derived Factor: Inhibition of Phosphorylation of Insulin Receptor (IR)/IR Substrate (IRS), Osteogeneration from Adipocytes, and Increased Levels Due to Doxorubicin Exposure

**DOI:** 10.3390/pharmaceutics15071960

**Published:** 2023-07-15

**Authors:** Isobel C. Jones, Revathy Carnagarin, Jo Armstrong, Daphne P. L. Lin, Mia Baxter-Holland, Mina Elahy, Crispin R. Dass

**Affiliations:** 1 Curtin Medical School, Curtin University, Bentley, WA 6102, Australia; 2 School of Medicine, University of Notre Dame, Fremantle, WA 6160, Australia; 3 Dobney Hypertension Centre, School of Medicine—Royal Perth Hospital Unit, Faculty of Medicine, Dentistry & Health Sciences, University of Western Australia, Perth, WA 6009, Australia; 4 School of Pharmacy, Curtin University, Bentley, WA 6102, Australia; 5 School of Pharmacy and Biomedical Sciences, Curtin University, Bentley, WA 6102, Australia; 6 School of Medical Sciences, University of New South Wales, Kensington, NSW 2052, Australia; 7 Curtin Health Innovation Research Institute, Curtin University, Bentley, WA 6102, Australia

**Keywords:** PEDF, metabolism, insulin, differentiation, doxorubicin

## Abstract

Objectives: Pigment epithelium-derived factor (PEDF) has been recently linked to insulin resistance and is capable of differentiating myocytes to bone. We examined in more detail the intricate signalling of the insulin pathway influenced by PEDF in skeletal myocytes. We tested whether this serpin is also capable of generating de novo bone from adipocytes in vitro and in vivo, and how the anticancer drug doxorubicin links with PEDF and cellular metabolism. Methods and key findings: We demonstrate that PEDF can inhibit phosphorylation of insulin receptor (IR) and insulin receptor substrate (IRS) in skeletal myocytes. PEDF constitutively activates p42/44 MAPK/Erk, but paradoxically does not affect mitogenic signalling. PEDF did not perturb either mitochondrial activity or proliferation in cells representing mesenchymal stem cells, cardiomyocytes, and skeletal myocytes and adipocytes. PEDF induced transdifferentiation of adipocytes to osteoblasts, promoting bone formation in cultured adipocytes in vitro and gelfoam fatpad implants in vivo. Bone formation in white adipose tissue (WAT) was better than in brown adipose tissue (BAT). The frontline anticancer drug doxorubicin increased levels of PEDF in a human breast cancer cell line, mirroring the in vivo finding where cardiac muscle tissue was stained increasingly for PEDF as the dose of doxorubicin increased in mice. PEDF also increased levels of reactive oxygen species (ROS) and glutathione (GSH) in the breast cancer cell line. Conclusions: PEDF may be used to regenerate bone from adipose tissue in cases of trauma such as fractures or bone cancers. The increased presence of PEDF in doxorubicin-treated tumour cells need further exploration, and could be useful therapeutically in future. The safety of PEDF administration in vivo was further demonstrated in this study.

## 1. Introduction

Pigment epithelium-derived factor (PEDF) is a multifunctional serpin involved in insulin resistance, thereby having a substantial role to play in metabolism [1,2,3]. Encoded by serpin F1 (family F, member 1), this gene encodes a member of the serpin family that does not display the serine protease inhibitory activity shown by many of the other serpin proteins. PEDF is known to inhibit insulin signalling in skeletal myocytes, regulating glucose homeostasis through Akt/PKB-dependent and independent pathways [3]. In this study, we delve deeper into the inner mechanisms of PEDF perturbing insulin signalling, providing a few novel molecular aspects of PEDF’s influence on insulin signalling, specifically the role of phosphorylation on insulin receptor (IR) and insulin receptor substrate 1 (IRS).

PEDF is capable of bone formation via differentiation of pre-osteoblasts to osteoblasts [4], and is able to induce bone formation in skeletal muscle when embedded in various implant devices (nanoparticles [5], gelfoam discs [6], or alginate beads [7]). When mice were co-administered insulin, bone osteogeneration in muscle was dampened, confirmed by lack of expression of bone markers in the muscle and surrounding adipose tissue. The latter prompted us to see if PEDF could be influential in transdifferentiating adipose tissue to bone tissue, in vitro as well as in an animal model, as doing the same in muscle tissue would not be practicable clinically. Both white and brown adipose tissues were examined. Proper bone health is lacking in diabetes patients [8], and the ability to use a patient’s own adipocytes to generate new bone would be a substantial step forwards in ensuring better bone health in diabetics. We also ensured that PEDF was not dampening the metabolic activity of these cells whilst promoting bone formation. In vivo, we monitored whether PEDF administration caused histological changes to the pancreas, small intestine, muscle, and skin adjacent to the treatment site.

Moreover, previously we have seen PEDF to be able to decrease the toxicity of the frontline anticancer drug doxorubicin, very active against certain cancers such as osteosarcoma, in such tissues such as the heart, small intestine and testis [9]. Although Dox is widely administered as a chemotherapeutic, problems with its severe cardiotoxicity remain a limiting factor in its applicability [10]. PEDF is a multifunctional protein with both antitumour and cardioprotective properties and represents a potential co-therapeutic option for the administration of Dox. To this end, we evaluated whether endogenous levels of PEDF increased due to exposure of cells and tissues to doxorubicin, and whether exogenous PEDF attenuates reactive oxygen species (ROS) and GSH levels in tumour cells. In addition, we tested whether PEDF, known for its ability to reduce cell cycling [11], reduces cell cycling in normal cells.

## 2. Materials and Methods

### 2.1. Materials

Recombinant PEDF and PEDF antibody was purchased from MD Bioproducts (Bethesda, MD, USA). Insulin and foetal bovine serum (FBS) were from Gibco (Fort Worth, TX, USA). Ninty-six-well plates and other tissue culture plasticware were purchased from Interpath (Heidelberg West, VIC, Australia). Dulbecco’s Modified Eagle’s Medium, penicillin/streptomycin and trypsin were from Life Technologies. Skeletal Muscle Myoblast Cell Media—Clonetics™ and SkBM™-2 BulletKit™ from Lonza (Walkersville, MD, USA). The IRβ, phospho-IRβ (tyr1162/1163), IRS-1, phosphor-IRS1 (tyr 632), and Glut4 antibodies were from Santa Cruz Biotechnology, Inc. (Dallas, TX, USA). The other antibodies such as IRS, pS307 and pS619IRS, *α*-actinin, *β*-tubulin, *p*-p42/44MAPK (Thr202/Tyr204), *p*-p38 MAPK (Thr180/Tyr182), and secondary anti-rabbit IgG, HRP-linked antibody were from Cell Signaling Technology (Beverly, MA, USA). The fluorescent Alexa Fluor^®^ 594 F(ab’)2 goat anti-rabbit IgG and Pierce 660nm protein assay were from ThermoFisher Scientific (Waltham, MA, USA). ECL Prime™ immunoblotting detection reagent kit was from GE Healthcare Amersham (Melbourne, VIC, Australia). Vectastain ABC kit purchased from Vector Labs (Burlingame, CA, USA). Cell-Titer Blue™ cell viability assay was from Promega (Madison, WI, USA). Cell lines were from the American Tissue Culture Collection, ATCC (Manassas, VA, USA), except the human skeletal muscle myoblasts (HSMMs) which were from Lonza (Walkersville, MD, USA) and were used within 10 passages from receipt. Cells were cultured in Dulbecco’s Modified Eagle Medium (DMEM) supplemented with 10% *v*/*v* foetal bovine serum (FBS) and 1% antibiotics and antimycotics at 37 °C within a humidified 5% CO_2_ chamber. 3T3-L1 cells were differentiated to adipocytes using standard protocol prior to treatment [12]. Penicillin/ streptomycin, Dulbecco’s Modified Eagle Medium (DMEM), Doxorubicin (Dox), trypsin, paraformaldehyde, bovine serum albumin (BSA), saponin, glycerol, Triton X100, dimethyl sulfoxide (DMSO), Tris-ethylenediaminetetraacetic acid (EDTA), foetal bovine serum (FBS), haematoxylin, eosin, 3,3′-Diaminobenzidine tablets (DAB), xylene and absolute ethanol were from Sigma-Aldrich (St Louis, MO, USA). The ABC kit (for immunocytochemistry (ICC) and immunohistochemistry (IHC)) was from Polyclonal goat anti-rabbit biotinylated antibody and normal goat serum (NGS) were purchased from Dako (Mulgrave, VIC, Australia). Phosphate buffered saline (PBS), ROS and glutathione (GSH) assay kit were from ThermoFisher Scientific (Malaga, WA, Australia).

### 2.2. Animal Ethics

All animal experiments complied with the ARRIVE guidelines and were carried out in accordance with the U.K. Animals (Scientific Procedures) Act, 1986. Stem cells were harvested from mice of both sexes, and the implant study was performed in female mice. Gender was deemed not to have an effect on either of these two studies using animals.

### 2.3. Mesenchymal Stem Cell (MSC) Isolation and Culture

The bone marrow stromal cell (BMSC) isolation protocol was prior approved by the Victoria University Animal Experimentation Ethics Committee, approval number 16-10, approved 1 July 2012). MSCs were isolated from the marrow resident in the long bones of 6-week-old Balb/c mice and trilineage differentiation potential of the cells to chondrogenic, adipogenic and osteogenic pathways were tested for lineage confirmation [7].

### 2.4. Viability Assay

The proliferation of C2C12 and HSMM cells following PEDF treatment was assessed using the Cell Titre Blue™ assay fluorimetrically, 560 nm_Ex_/590 nm_Em_ (Promega, Madison, WI, USA) according to the manufacturer’s instructions using a multimode Plate Reader (Enspire Perkin Elmer, Waltham, MA, USA).

### 2.5. Immunoblotting

Immunoblotting was performed on lysates from treated cells as before [3]. Blots were stripped and reprobed with antibodies to housekeeping proteins. Antibodies were visualised with chemiluminescent reagent (ECL Prime, GE Healthcare Amersham, Melbourne, Australia), and images acquired with a Gel-Doc EZ instrument (Biorad, Gladesville, NSW, Australia).

### 2.6. Fluorescence Immunocytochemistry

Immunofluorescence experiments were performed as before (Carnagarin et al. 2016) in mice and human skeletal myocytes. Cells were incubated with primary antibodies at 1:250 dilution and 1:2000 secondary antibody. Images were observed under Olympus IX51 fluorescent microscope (Olympus Life Science Solutions, Breinigsville, PA, USA). The GLUT4 translocation images were analysed by measuring the fluorescent intensity in the perinuclear region using Fiji/Image J2 software (https://imagej.net/downloads; accessed 6 June 2023).

### 2.7. Von Kossa Staining

Treated cells were subjected to von Kossa staining as before [13]. Post-fixing the cells with 4% paraformaldehyde for 30 min, mineralised nodules were stained with 5% silver nitrate and the plate placed under light for 30 min. Wells were rinsed once with water, treated with 5% sodium thiosulphate solution for 5 min, then washed with water once more, prior to images being acquired.

### 2.8. Gelfoam Binding and Release

Gelfoam blocks were aseptically cut from the (5 mm × 5 mm × 7 mm) supplied sheet, and soaked in 100 μL of 100nM PEDF (in sterile water) for 1 h in microfuge tubes. Imbibed blocks (12 units) were removed and the remnant PEDF solution kept aside for PEDF ELISA. The imbibed blocks were placed in 3 separate tubes, each tube containing 4 blocks and 300 μL of PBS, pH 7.4 buffer. At each timepoint, tubes were gently centrifuged (10× *g*), then supernatants (100 μL) removed for ELISA assay. Blocks were then incubated until the next timepoint.

### 2.9. Gelfoam Implantation Study

For the implantation assay, approval was obtained from the Curtin University Animal Ethics Committee (approval number 2013-21, approved 1 July 2013) prior to experimentation. Mice were induced in an anaesthetic chamber with a mixture of oxygen (2–3 L/min) and isoflurane (4%). Gelfoam (5 mm × 5 mm × 7 mm) was inserted into the abdominal fat pocket using dental tweezers for the control group (*n* = 8 mice/group) and gelfoam with 100 nM PEDF for the test group (*n* = 8 mice/group). This process was repeated for the back fatpad of each mouse. Mice were monitored closely after surgery until full recovery and were permitted unrestricted movement within their cages, with food and water, for 8 weeks. No observable differences were seen between the health of the mice in the treatment (PEDF) and control groups. At the end of the eight weeks, the implants and surrounding tissues were harvested. Additionally, part of the pancreas and small intestine was also harvested. Haematoxylin and eosin staining of the tissue samples was conducted, and images acquired using an Olympus BX51 upright light microscope (Breinigsville, PA, USA) with CellSens standard software, version 2.0 (Breinigsville, PA, USA) to capture images.

### 2.10. PEDF Detection in MDA-MB-231 Cells Exposed to Doxorubicin (Dox)

PEDF levels in cells was assessed using immunocytochemistry (ICC), as routinely performed in our lab [11]. Cultured human triple negative (ER^-^/PR^-^/HER2^-^) breast cancer MDA-MB-231 cells were trypsinised and seeded at a density of 5000 cells/well in 10% FBS DMEM into 96-well plates. Cells were incubated for 24 h to allow adherence before the assay. Following this, 0, 0.1 and 1 μM Dox treatments were applied, and cells were incubated for 24 h. Cells were then fixed in 4% paraformaldehyde and permeabilised in 0.3% saponin. Next, 2% NGS/0.25% BSA/0.1% saponin was applied for blocking. Primary PEDF antibody (1:500 diluted in PBS) was applied overnight, followed by secondary antibody (1:1000 diluted in PBS) for 30 min. Avidin/biotinylated horseradish peroxidase (HRP) mixture from the ABC kit was applied per the manufacturer’s instructions. Cells were incubated in DAB solution and washed, then mounted in 100% glycerol. Wells were then imaged using a Nikon Eclipse Ti inverted microscope (Melville, NY, USA) and CellSens imaging software. 

### 2.11. PEDF Detection in Mouse Cardiac Tissue

PEDF levels in the cardiac tissue of mice treated with Dox were assessed using immunohistochemistry (IHC). Balb/c mice were treated with 0.1 mL injections of 0 mg/kg, 1 mg/kg and 3 mg/kg doses of Dox 3 times weekly for one month (AEC code 10/12) [9]. After the injection schedule, the mice were euthanised, and the heart was harvested and processed. Organs were placed in 4% paraformaldehyde and embedded in paraffin. Organ blocks were then sectioned at 5 μm and fixed on slides. Tissues were deparaffinised by two washes of 100% xylene for 3 min each. To rehydrate the sections, slides were washed in decreasing concentrations (100%, 100%, 95%, 70% and 30% ethanol, each for 3 min). Antigens were retrieved by exposing the slides to high pH Tris-EDTA buffer for 12 min at 95 °C [14]. Endogenous peroxidase activity and non-specific binding were blocked by incubating slides in 0.3% hydrogen peroxide for 30 min, and 10% normal goat serum and 0.25% BSA for 30 min, respectively. Primary antibody was applied at a 1:500 dilution in PBS for 3 h at room temperature (RT). The secondary antibody was applied at a 1:1000 dilution in PBS for 30 min at RT. Avidin/biotinylated HRP mixture from the ABC kit was added as per manufacturer’s instructions for 30 min. DAB was then added and incubated for 3 min. Slides were dehydrated by washing with increasing concentrations of ethanol (30%, 70%, 100% and 100%, each for 3 min) and then cleared with 100% xylene for 1 min. Slides were mounted with DPX (Sigma-Aldrich, Castle Hill, VIC, Australia) and imaged.

### 2.12. Measurement of ROS and GSH

Cultured cells were trypsinised and seeded at a density of 5000 cells/well in 10% FBS DMEM into 96-well plates. Cells were incubated for 24 h to allow adhesion before the assay. Cells were then treated with 0, 0.1 or 1 μM Dox ± 100 nM PEDF for the ROS measurement assay and 5 μM Dox ± 100 nM PEDF for the GSH measurement assay. Plates were incubated for another 24 h, except for the 5 μM GSH measurement plate, which was incubated for 1 h to avoid excessive cell death. After incubation, the ROS measurement wells had 5 μM H2DCFDA added, and the GSH measurement wells had 6 μM CMFDA added and incubated for another 1 h. Plates were then analysed at 485 nm excitation and 535 nm emission using an EnSight multimode microplate reader (Perkin Elmer, Akron, OH, USA). The contents of the wells were then removed and replaced with fresh medium. Next, microphotographs of wells were taken using a Nikon Eclipse Ti inverted microscope and CellSens imaging software (Olympus, Notting Hill, VIC).

### 2.13. Statistical Analysis

Results are presented as mean ± standard deviations. Statistical significance was determined using one way or two-way analysis of variance or a *t*-test on MS-Excel (Redmond, WA, USA). A *p* < 0.05 was taken to indicate significance for all assays. Individual number of replicates (*n*) for each study is given in the captions to all relevant figures.

## 3. Results

### 3.1. PEDF Impairs Metabolic Signalling via IRS-1 and Constitutively Activates p42/44 MAPK/Erk Kinase

**Figure 1:**
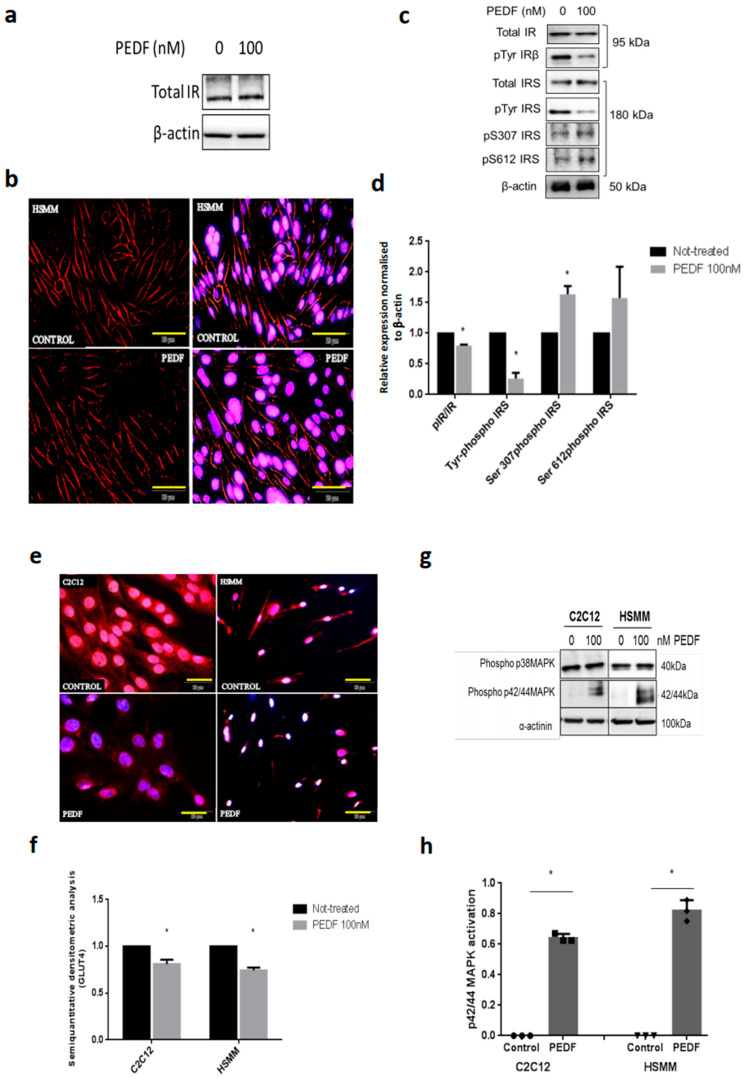
PEDF impairs metabolic insulin signalling in HSMMs, impairs Glut4 translocation and activates phosphor p42/44 MAPK with no effect on proliferation. (**a**) Analysis of molecular signalling events modulated by PEDF on HSMMs myocytes by immunoblotting revealed that PEDF (over 24 h) had no effect on the total insulin receptor (IR). (**b**) Immunofluorescence results showing the absence of effects of PEDF on total IR in HSMMs. Scale bar = 50 µm. **Top**, control (untreated), **bottom**, PEDF-treated. (**c**) Immunoblot analysis showing PEDF inhibited tyrosine phosphorylation activation of insulin receptor (IRβ) and insulin receptor substrate (IRS) and induced IRS serine phosphorylation in HSMMs. (**d**) Relative expression analysis of selective IR signalling molecules in HSMMs. (**e**) Immunofluorescence data showing PEDF treatment inhibited GLUT4 translocation in C2C12 and HSMM. Scale bar = 50 µm. (**f**) Semiquantitative densitometric analysis of the fluorescent intensity of GLUT4 at the perinuclear region analysed with Fiji software (NIH). Values are mean ± SD (*n* = 3), * *p* < 0.05. (**g**) MAPK signalling was not interrupted by PEDF treatment but acute activation of p42/44 kinase/Erk was noticed. (**h**) Relative expression analysis of p42/44 signalling molecule.

To study the molecular events associated with PEDF effects in detail, we employed the murine myoblast cell line, C2C12 along with the human equivalent, HSMMs. PEDF did not perturb total IR levels nor IR localisation in cells (Figure 1a,b), but reduced tyrosine phosphorylation of both IR and IRS1 as we have demonstrated previously [3], and, as shown here seminally, induced serine phosphorylation of IRS1 in C2C12 cells (Figure 1c,d). PEDF reduced GLUT4 translocation in both cell lines, C2C12 and HSMM (Figure 1e,f). PEDF induced constitutional activation of p42/44 MAPK/Erk in the skeletal myocytes (Figure 1g,h).

### 3.2. PEDF Did Not Perturb Viability of Various Cell Lines and Continued to Induce Bone Formation under Hyperglycaemic Conditions

**Figure 2:**
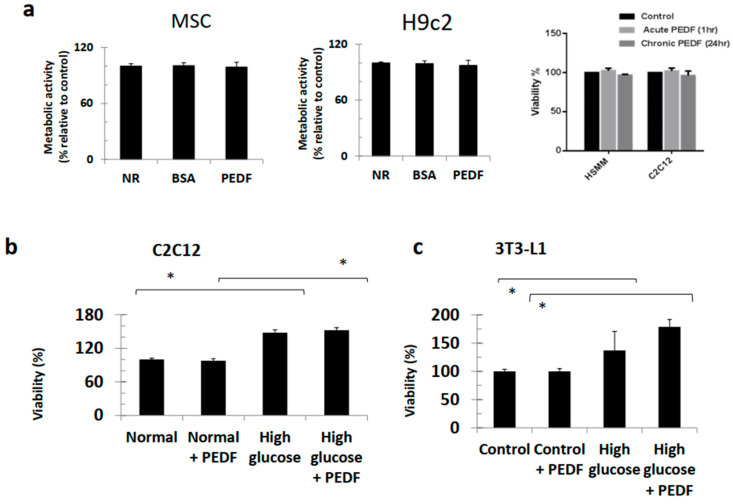

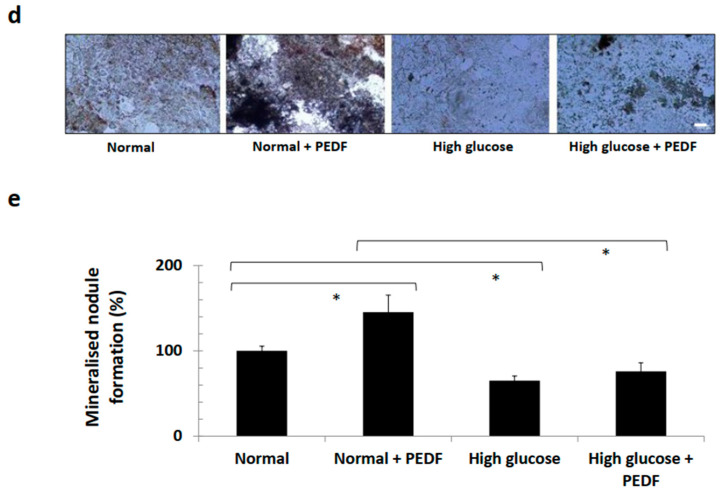
PEDF does not perturb viability in either normo- or hyperglycaemic conditions. (**a**) PEDF treatment did not have any effect on cell survival indicating uninterrupted mitogenic signalling in both C2C12 cells and HSMMs, *p* < 0.001. (**b**) PEDF had no effect on viability in C2C12 cells in both normo- and hyperglycaemic conditions. (**c**) PEDF had no effect on viability in 3T3-L1 cells in both normo- and hyperglycaemic conditions. (**d**) Photomicro-graphs of von Kossa results showing nodule formation (brown deposits). Scale bar = 100 μm. (**e**) Graphed von Kossa results showing decrease in nodule formation under hyperglycaemic conditions, and the increase in nodule formation in the presence of PEDF. Scale bar = 50 μm. * *p* < 0.05.

Metabolic activity (viability) of PEDF-treated MSC and H9c2 cells was similar to the control (untreated) condition (Figure 2a). Effects of PEDF on viability were not dissimilar between 1 h (acute) or 24 h (chronic) conditions in HSMM and C2C12 cells. Under hyperglycaemic conditions, viability of C2C12 and 3T3-L1 cell lines increased 50% on average when compared to normoglycaemic conditions (Figure 2b,c). Importantly, PEDF did not affect viability of either line under hyperglycaemic conditions. However, PEDF increased bone nodule formation in 3T3-L1 cells, approximately 50% in normo- and 20% under hyperglycaemic conditions (Figure 2d,e). High glucose conditions reduced bone nodule formation compared to normoglycaemic conditions.

### 3.3. PEDF Implanted in Adipose Tissue (Brown and White) can Induce De Novo Osteogeneration, with Lack of Harmful Effects to Surrounding Tissues

**Figure 3:**
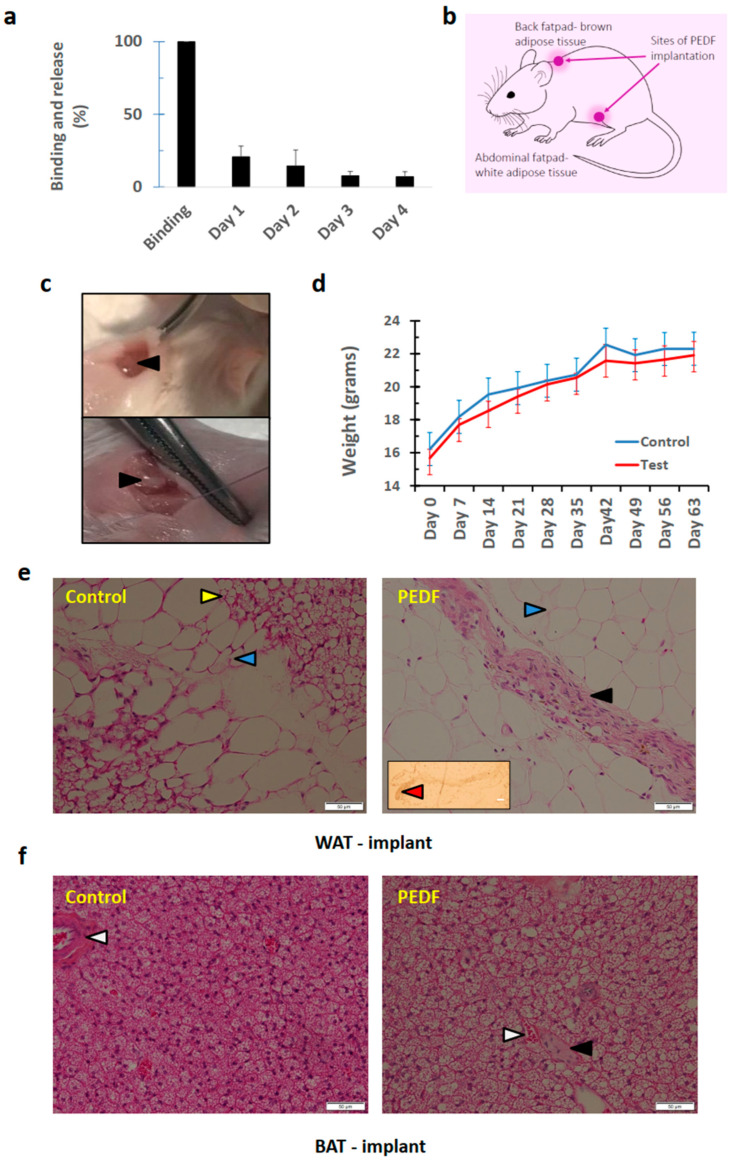
PEDF is able to transdifferentiate adipose cells to bone tissue. (**a**)**.** PEDF binding to and release from Gelfoam. (**b**)**.** Schematic showing sites of Gelfoam implantation. (**c**)**.** Photos showing fatpad (**top**) and implant position (**bottom**) indicated with arrowheads. (**d**)**.** Mice were weighed to assess any signs of gross toxicity due to the implants. (**e**)**.** Photomicrographs of the white adipose tissue (WAT) showing signs of osteoid tissue being formed. *Insert*, non-mineralised osteoid tissue. *Arrowheads: yellow*, Gelfoam implant; *blue*, adipose tissue; *black*, osteoid; *red*, lack of von Kossa staining. (**f**)**.** Photomicrographs of the brown adipose tissue (BAT) showing signs of osteoid tissue being formed. *Arrowheads: white*, blood vessel; *black*, osteoid. *Scale bar* = 50 μm. *n* = 8 mice/group.

We found that the Gelfoam was able to bind PEDF efficiently (Figure 3a). Release per diem decreased from 20 to 6% over the 4 days of study in PBS in vitro. In the in vivo study, implants were introduced at two sites, brown and white adipose fat (BAT and WAT, respectively) as indicated in Figure 3b,c. Mouse weights between the two cohorts did not differ (Figure 3d). In some cases, implants are assimilated into the surrounding tissues without a clear junction of where implant ends and solid tissue begins, indicating that cells have infiltrated and successfully grown within the implant. In the WAT of control mice, the implants both with and without PEDF were incorporated into the surrounding adipose tissue without impacting on the health of the surrounding cells. In the PEDF implants, regions containing osteoid tissue were seen, but not in the control group (Figure 3e). A similar finding was noted in BAT, where osteoid tissue was identified (Figure 3f). However, PEDF did not appear to generate the same level of osteogenic tissue formation within the BAT as it did for the WAT. There is a clear distinction between the globular adipocytes and the tissue that is present amongst them, which was not present in the control group tissues, that is without PEDF. Formation of osteoids occurred at multiple sites and vasculature was present amongst the newly formed osteogenic tissues. The cytoplasm of these cells were stained a darker pink due than those of the surrounding adipocytes.

Interestingly, there were more vessels noted in the PEDF cohort in both WAT and BAT implant areas (Supplementary Figure S1a,b). PEDF appears to induce histological changes in pancreatic tissues (Supplementary Figure S1c). In the control tissues, the pancreatic islets are separated from the exocrine cells. Islets contain the active cells of the pancreas, such as α, β and δ cells, pancreatic polypeptide (PP) cells, and ε cells. The islets in the test mice (those that received PEDF) were characterised by less-defined borders. The exocrine tissues surrounding the islets in the test mice also look to have changed slightly, with greater eosinophilic granule presence in the PEDF cohort. In the small intestine (Supplementary Figure S1d), it was noted that PEDF reduced the quantity of goblet cells present in the villi. There were no differences in the frequency or size of blood vessels though. In skeletal muscle, there was no difference between the cohorts (Supplementary Figure S1e). Similarly, no differences were noted between cohorts for skin (Supplementary Figure S1f).

### 3.4. PEDF Levels Increased in Human Breast Cancer Cell Line Exposed to Doxorubicin

**Figure 4:**
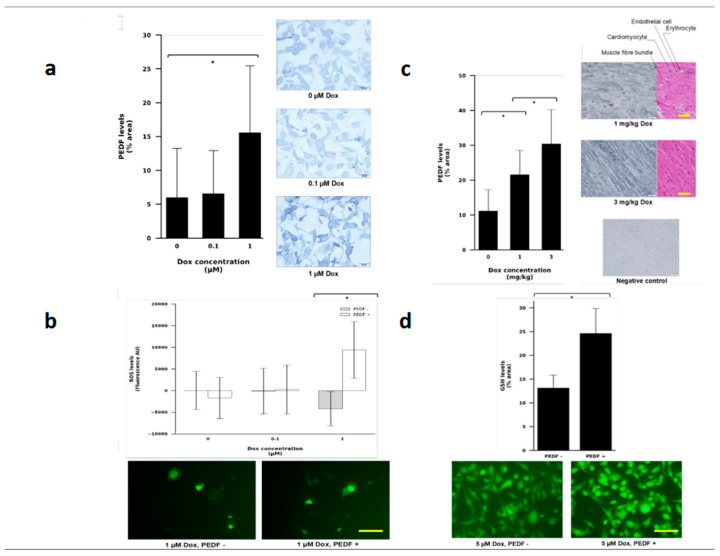
PEDF is upregulated in breast cancer cells (MDA-MB-231) treated with doxorubicin. (**a**)**.** Percentage of dark-stained area representative of PEDF levels. Data are represented as mean ± standard deviation, * *p* < 0.05, *n* = 4, scale bar = 50 μm. (**b**)**.** Fluorescent intensity of cells indicating levels of ROS. Data are represented as mean ± standard deviation, * *p* < 0.05, *n* = 4, scale bar = 50 μm. (**c**)**.** Percentage of dark-stained tissue area indicative of relative levels of PEDF. Ten representative images were taken across the replicates of each condition to calculate average percentage of PEDF staining. Data are represented as mean ± standard deviation, * *p* < 0.05, *n* = 6 (0 and 1 mg/kg) and *n* = 3 (3 mg/kg), scale bar = 20 μm. H&E demonstrate representative images of tissue from 2 conditions. (**d**)**.** Fluorescent intensity of cells indicating levels of GSH. 5 representative images were taken across the replicates of each condition to calculate average percentage of staining intensity. Data are represented as mean ± standard deviation, * *p* < 0.05, *n* = 4, scale bar = 50 μm.

To investigate the impact of Dox administration on endogenous PEDF levels, we examined PEDF levels in response to Dox addition to TNBC cell line, MDA-MB-231. Compared to the 0 μM Dox control group, 0.1 μM Dox did not significantly increase levels of PEDF (Figure 4a). At 1 μM Dox, PEDF levels significantly increased compared to 0 μM Dox treatment (*p* = 0.0039). There were more spindle-shaped, darker DAB-stained cells in the 1 μM Dox treatment group than in the other two groups. To examine the influence of PEDF on intracellular ROS levels during Dox treatment, MDA-MB-231 cells were treated with increasing concentrations of Dox for 24 h in the absence or presence of exogenous PEDF. The fluorescent intensity measured by the microplate reader showed that PEDF had no significant effect on ROS levels in 0 and 0.1 μM Dox treated cells (Figure 4b). At 1 μM Dox concentration, however, PEDF addition significantly increased levels of intracellular ROS (*p* = 0.0118). In the absence of PEDF, the cells treated with 1 μM Dox displayed a decrease in ROS levels. This finding was, however, not statistically significant compared to the 0 μM Dox group. In the representative images taken under the microscope, the cell nuclei in the PEDF group were more intensely fluorescent than the nuclei of cells untreated group.

To further investigate the effect of Dox administration on PEDF levels, we assayed heart tissue samples of mice treated with Dox for PEDF levels using IHC. Dox administration resulted in PEDF upregulation, with a significant increase between 0 and 1 mg/kg (*p* = 0.0022) and 1 and 3 mg/kg (*p* = 0.0319) (Figure 4c). The 3 mg/kg tissue had the highest PEDF staining than the 1 mg/kg and 0 mg/kg tissues. To further examine the effect of PEDF on the redox balance in cells treated with Dox, MDA-MB-231 cells were treated with Dox for 1 h in the presence and absence of exogenous PEDF and assayed for GSH levels. The fluorescent intensity of the PEDF group was significantly greater than the Dox-only group (Figure 4d), showing that PEDF significantly increased GSH levels (*p* = 0.0319). In the representative images taken under the microscope, cells in the PEDF^+^ group had a brightly fluorescing nucleus and less visible cytoplasm.

## 4. Discussion

In this present study, PEDF induced serine phosphorylation of IRS1 in skeletal myocytes, and induced constitutional activation of p42/44 MAPK/Erk in the skeletal myocytes. Insulin binds with IR and causes phosphorylation of the tyrosine residues of IR which elicits a downstream phosphorylation cascade. The tyrosine phosphorylated substrates bind several Src homology 2 (SH2) proteins linking the tyrosine kinase to activation of two major signalling pathways, one involving a ras/mitogen-activated protein kinase (MAP kinase) cascade and the other involving IRS/phosphatidylinositol 3-kinase (PI3-kinase) which causes the biological actions of insulin [15]. IRS1 is a key adapter molecule that further propagates the downstream metabolic signal through phosphorylation activation of protein kinase B (PKB). Insulin-resistant states have been associated with reduced insulin-stimulated tyrosine phosphorylation of IRS-1 or with serine-phosphorylation of IRS-1 or impaired PKB downstream signal propagation [16,17].

Encouraged by our previous findings that PEDF can induce osteoregeneration from myocytes and muscle tissue in mice [5,7,18], we embarked on our next study examining whether PEDF could increase bone nodule formation in adipocytic cells. Clinically, adipose tissue would be a better site for generation of new bone tissue than muscle given its abundance, lack of abundant innervation (and therefore lesser pain sensations), and the fact that this tissue can be harvested without much impact on patient health (as occurs in liposuction). When PEDF-imbibed gelfoam was implanted into two fat deposits in mice (WAT and BAT), nodule formation was noted histologically. Von Kossa staining indicated that the nodules were not mineralised (or calcified) as yet, signifying early-stage bone formation. The difference between WAT and BAT is their function; WAT is primarily an energy bank, storing triglycerides during excessive energy intake, and BAT is more ‘metabolically active,’ has a much higher level of mitochondria and functions primarily to burn calories to generate heat [19]. BAT is associated with more metabolically healthy phenotypes, whereas higher levels of WAT promote insulin resistance, type II diabetes mellitus, and metabolic inflammation [20]. It would appear that PEDF and WAT act synergistically; however, the actions of PEDF antagonise those of BAT. The two types of adipose tissue are not static, and WAT is able to convert into BAT-like cells [21]. In addition, the cells have different lineage origins, develop from different areas of the mesoderm, and require different factors for terminal differentiation [22].

For example, precursor cells treated with BMP-2 or BMP-4 became WAT whilst those treated with BMP-7 and PR domain containing 16 (PRDM16) became BAT [23]. PEDF’s mechanisms of inducing osteogenic tissue formation include activating MAPK/ERK and alkaline phosphatase (ALP) pathways [24], inhibition/promotion of key mineralisation factor such as sclerostin and dentin matrix protein 1 (DMP-1) [25], and activation of Wnt pathway to promote a shift in cell fate to the osteogenic lineage [26]. With the differences between the types of adipose tissue in mind, the greater level of osteogenic tissue formed within WAT could be attributed to the tissue being characteristically more responsive to the influence of PEDF, or the progenitor cells present within WAT are more easily converted toward the osteogenic lineage than those of BAT.

Interestingly, despite PEDF being an extremely potent angiogenesis inhibitor [27] capable of downregulating VEGF [4], no changes in the appearance or number of vasculature formation between test and control tissues were observed. Angiogenesis inhibition may be a part of PEDF’s metabolic disruption activity. Adipose tissue, particularly BAT, is very highly vascularised and angiogenesis within this tissue has been shown to play a role in the homeostasis of adiposity [28,29]. Stimulation of angiogenesis in BAT has been hypothesised to be beneficial against obesity due to its facilitation of energy expenditure and increase in metabolic rate [28,29,30]. However, the same beneficial effects are not seen for WAT; vessels supply this tissue with nutrients, oxygen, stem cells, plasma enriched with growth factors that promote WAT expansion and thus obesity, plus facilitate the infiltration of the immune cells associated with metabolic inflammation in the overweight and obese [29,31,32]. Angiogenesis inhibitors have been considered for use as obesity therapy, based on the knowledge that expansion of WAT relies on angiogenesis. This hypothesis has been proven positive in mice with the inhibitors endostatin, angiostatin, VEGFR2 and TNP-470 displaying the ability to reduce body weight or prevent the development of obesity in subjects prone to excessive weight gain [32,33,34]. Promisingly, we did note a decrease in vasculature in the WAT and BAT by PEDF in this study. One limitation of the present study is that the model did not full mineralisation of the bone, due to the shorter period of evaluation time. This opens up the possibility of future research to test this further in actual models where bone needs to be mature, example, fracture models. However, the positive findings here provide a reliable launchpad for such future studies.

We have previously shown that PEDF is safe to tissues in mice [35]. In the present study, histological differences between the pancreatic tissues of the control and test samples, particularly regarding the islets, were uncovered. Islets of mice that received PEDF were characterised by less-defined borders, a finding that has been associated with insulin resistance previously [36]. Previously, we have shown that PEDF increases secretion of insulin from islet cells by more than twofold, though when intact islets are incubated with PEDF, the increase is minimal over 24 h [37]. The exocrine tissues surrounding the islets in test mice in the current study also changed slightly, with the eosinophilic granules being more abundant in the PEDF group. This may mean that there is an increase in production and secretion of digestive enzymes in the pancreas when stimulated by PEDF, though this needs testing. Within the islets, there was a reduction in concentration of β-cells in test mice, with the nuclei appearing more sparse than those of the control group. The islets in test subjects appeared to branch out and be slightly more stellate than those in the control group, with mitotic cells, all indicative of early cell hyperplasia. In general, β-cell mass is adjusted dynamically via external demand during adult life [38]. When insulin resistance develops, β-cells of islets increase their mass to increase insulin production and secretion as a compensatory mechanism [39,40]. In the small intestine, a decrease in goblet cells noted in the PEDF cohort was interesting, there was no decrease in body weights of mice between the cohorts. With no discernible effects of PEDF on either the muscle or skin, overall, PEDF seems to be a safe protein to administer in vivo.

Our study demonstrated that PEDF increased ROS levels in MDA-MB-231 cells treated with 1 μM Dox. As mentioned, PEDF has been characterised as an antioxidant. In contrast to our results, it has been shown that PEDF can reduce advanced glycation end product-induced NADPH oxidase-dependent ROS generation in MCF-7 cells [41], though this was not in relation to Dox. Other studies have also identified PEDF-induced inhibition of NADPH oxidase-dependent ROS generation, including in human umbilical vein endothelial cells and H9c2 cells [32,42]. As far as we know, this is the first time that PEDF-induced ROS upregulation in Dox-treated tumour cells has been demonstrated. Since PEDF did not increase ROS in the 0 μM Dox group, PEDF’s pro-oxidant action in the MDA-MB-231 cells is most likely in response to Dox’s activity.

Interestingly, a recent study showed that Dox treatment inhibited glycolysis in in ovo grafted MDA-MB-231 cells, contributing to its cytotoxic action [43]. Previously, we have demonstrated that PEDF administered to skeletal muscle cells caused upregulation of NADPH oxidase-dependent ROS, which subsequently induced ROS-dependent induction of glycolysis without impacting mitochondrial respiration or cellular viability [3]. From this, it is possible that in our study, PEDF had a metabolic modulation role by inducing ROS-mediated glycolysis in response to Dox’s effect on cellular metabolism. MDA-MB-231 cells have been demonstrated to exhibit the Warburg effect, undergoing anaerobic glycolysis and producing lactate even in the presence of oxygen [44,45]. It would be interesting to see if, firstly, PEDF influences glycolysis in Dox-treated MDA-MB-231 cells and, secondly, if there is a difference in lactate accumulation in response to PEDF treatment. These data would be especially relevant when PEDF’s antimetastatic activity is considered since high lactate levels in cancers have been correlated with the chance of metastasis [46]. Interestingly, in cardiomyocytes undergoing oxygen/glucose deprivation, PEDF inhibited lactate accumulation by downregulating glycolysis [47].

A surprising observation from our study was that MDA-MB-231 cells treated with 1 μM Dox without PEDF showed decreased ROS levels. Although the decrease was not statistically significant, this finding conflicts with most of the literature, which concurs with the traditional redox cycling-induced ROS generation mechanism of Dox cytotoxic action [10]. Recent investigations have questioned the importance of ROS generation in Dox’s cytotoxic action. One study found that Dox could not increase ROS levels in human primary cardiomyocytes, lung cancer cells, neuroblastoma cells and leukaemia cells [48]. Moreover, another study showed that Dox semiquinone was undetected in the cytoplasm of Dox-sensitive lung cancer cells [49]. However, Dox semiquinone was present in Dox-resistance lung cancer cells. Moreover, increased oxidative stress was present in the Dox-resistant lung cancer cells, despite having higher cell viability. Another study found that retinoic acid-differentiated H9c2 cells had increased levels of superoxide dismutase and hydrogen peroxide resistance, despite possessing a higher sensitivity to Dox treatment than undifferentiated cells [50]. Since cell viability was also unaffected by Dox or PEDF-induced levels of ROS, our study supports the theory that the primary mechanism of Dox-induced cytotoxicity, at least in MDA-MB-231 cells, is not ROS generation. It is important to note that ROS detection via H2DCFDA is unable to detect all ROS variants, so it is still possible that Dox was able to significantly induce ROS which was not detected [51].

Taken together with the current literature, the results of our study elucidate the delicate interplay between PEDF’s activity and ROS and antioxidant generation in the context of Dox-treated tumour cells. PEDF plays a role in regulating these crucial cellular pathways in several cell types. Within this study and its similarities and conflicts with others, it has become evident that PEDF is not a protein that can be defined as having a consistently pro-survival or pro-death activity. Rather, PEDF’s activity is likely directed in response to specific cellular characteristics and activities which have not yet been defined, and very much linked to metabolic status of affected cells. Our study has also illustrated that Dox is a positive regulator of PEDF expression in both heart tissue and MDA-MB-231 cells, adding complexity to the role of PEDF in cells exposed to Dox. This study has generated a starting point for future work which should investigate the role of PEDF at the crossroads of metabolism and ROS generation in cancer cells and cardiomyocytes. Specifically, future research should seek to identify critical modulators of PEDF’s activity, how they are relevant across cell types and circumstances and how the concentrations of both Dox and PEDF influence this activity. Thus, this study has provided initial proof of the ability of PEDF to drive transdifferentiation of adipocytes towards osteoblasts, which could be used to treat bone trauma more efficiently and readily.

## Data Availability

The data underlying this article will be shared on reasonable request to the corresponding author.

## Supplementary Materials

The following supporting information can be downloaded at: https://www.mdpi.com/article/10.3390/pharmaceutics15071960/s1, Figure S1: PEDF is able to transdifferentiate adipose cells to bone tissue.
