# Supplementary material for: Pigment Epithelium-Derived Factor: Inhibition of Phosphorylation of Insulin Receptor (IR)/IR Substrate (IRS), Osteogeneration from Adipocytes, and Increased Levels Due to Doxorubicin Exposure
Source: Pharmaceutics. 2023 Jul 15;15(7):1960. doi: 10.3390/pharmaceutics15071960 (PMC10384968; doi:10.3390/pharmaceutics15071960)
Supplement: Supplementary file 1 [file pharmaceutics-15-01960-s001.zip › pharmaceutics-2448881-supplementary.pdf]

Supplementary Figure S1.

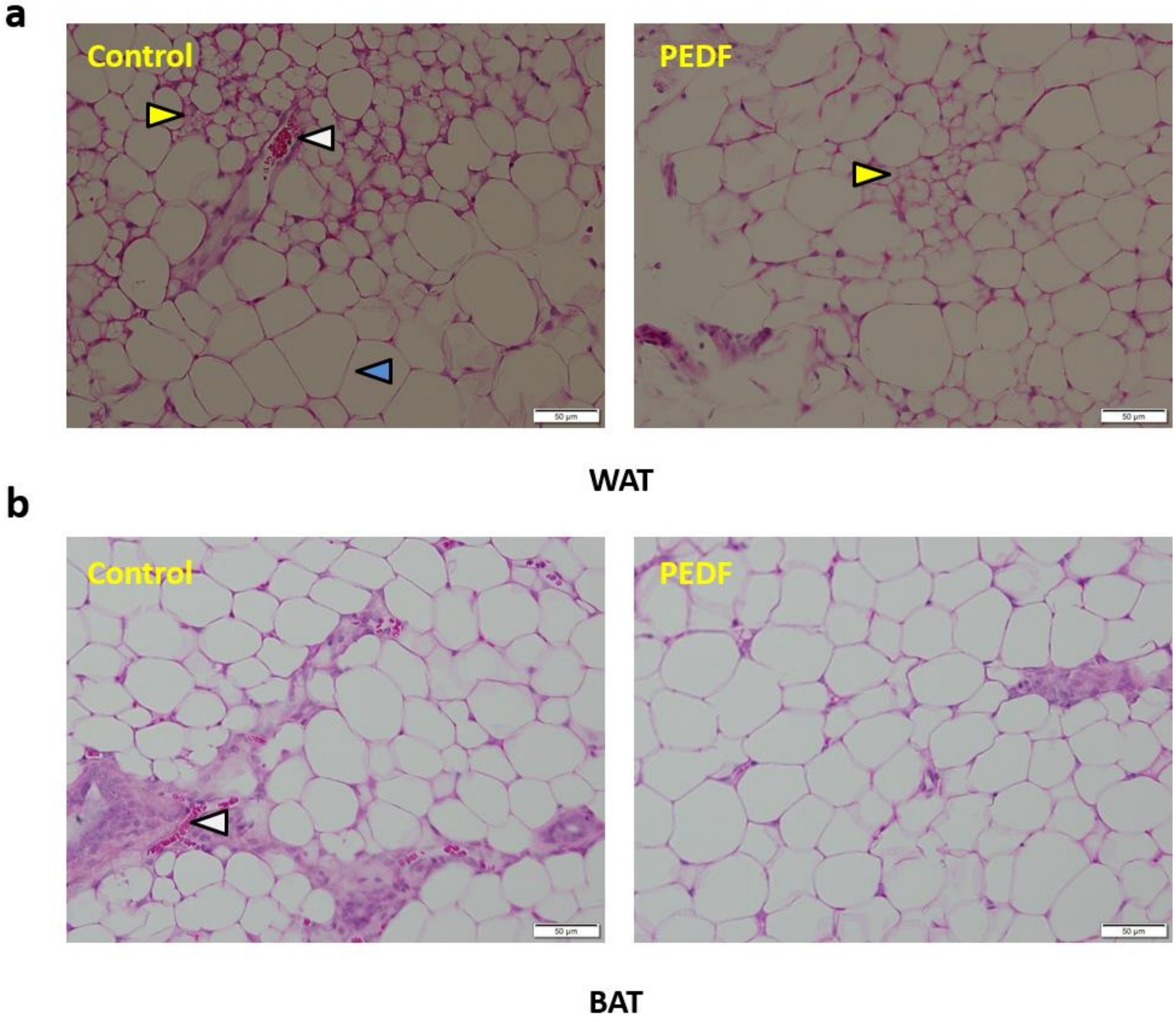

c

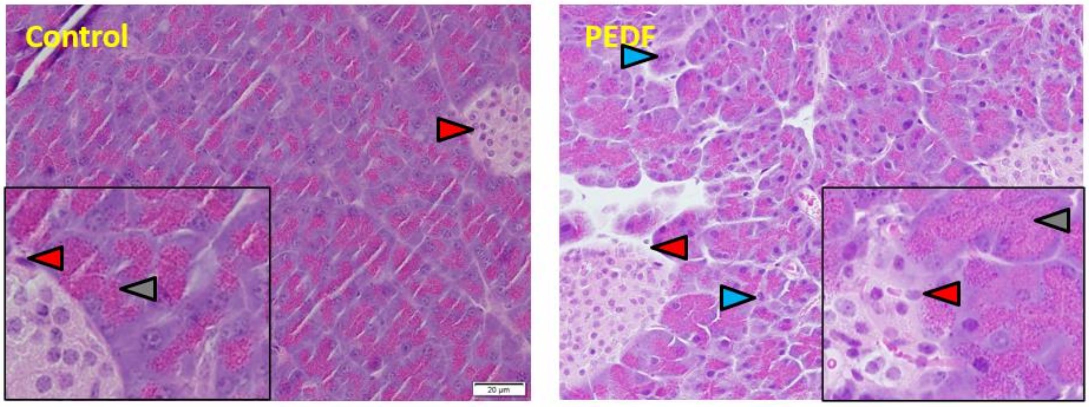

Pancreas

d

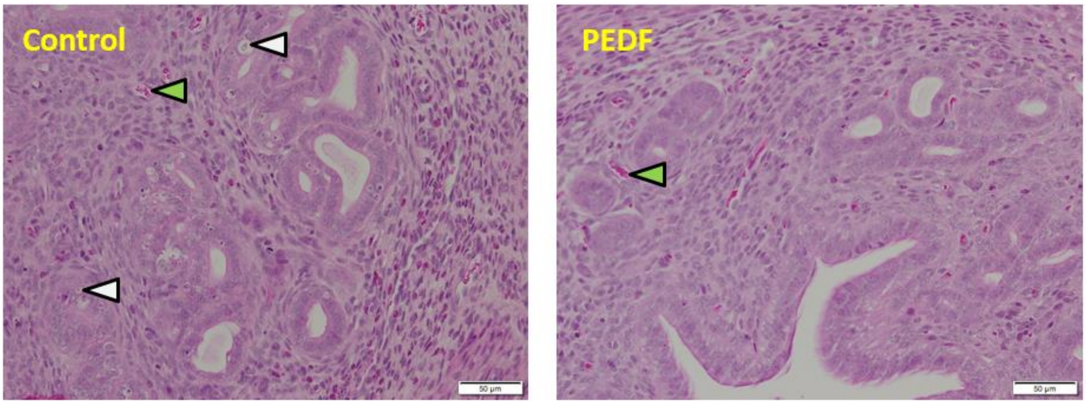

Small intestine

e

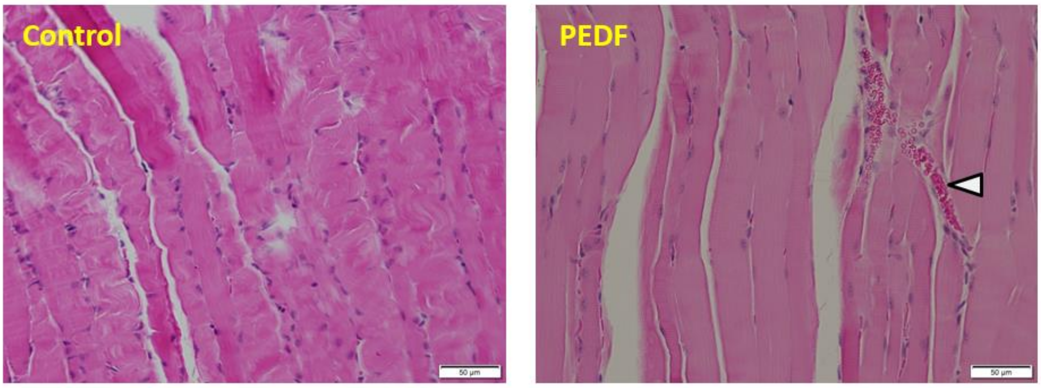

Muscle

f

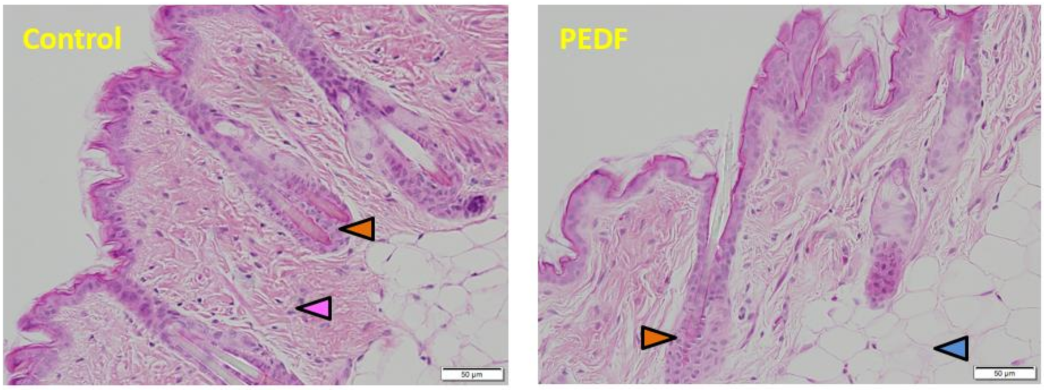

Skin

Supplementary Figure S1. PEDF is able to transdifferentiate adipose cells to bone tissue.

(a). Photomicrographs of the white adipose tissue (WAT) showing less blood vessels in the PEDF cohort. *Arrowheads: white*, blood vessel; *yellow*, Gelfoam implant remnant; *blue*, adipose tissue. **(b).** Photomicrographs of the brown adipose tissue (BAT) showing less blood vessels in the PEDF cohort. *Arrowheads: white*, blood vessel. **(c).** Photomicrographs of the pancreatic islet of Langerhans which appear to change around the boundary in the PEDF cohort. *Arrowheads: red*, islet of Langerhans; *grey*, exocrine secretory acini; *blue*, mitotic cells. **(d).** Photomicrographs of the small intestine showing nil differences between the cohorts. *Arrowheads: white*, goblet cell; *green*, blood vessel. **(e).** Photomicrographs of the skeletal muscle showing nil differences between the cohorts. *Arrowhead*, healthy blood vessel in the PEDF cohort. **(f).** Photomicrographs of the skin showing nil differences between the cohorts. *Arrowheads: orange*, hair follicle; *pink*, underlying dermal layer; *blue*, adipose tissue. *Scale bar* = 50 µm. *n* = 8 mice/group.
